# Medical and Philosophical News

**Published:** 1781-01

**Authors:** 


### SECTION III.

### Medical and Philosophical News.

THE Royal Society of Sciences at Copenhagen have propoſed the following prize queſtion: "Geneſin aeris puriſſimi, vulgo "dephlo-

" dephlogisticati, ex calcibus metallorum, vel " per se vel acido nitri saturatis, novis experi- " mentis ad majorem claritatis gradum per- " ducere, caput mortuum exactius examinare et " inquirere; anne eadem aeris species ope alio- " rum acidorum produci queat?" The prize is to be a gold medal, of the value of 100 rix dollars. The dissertations are to be written in Danish, French, Latin, or German, and sent (post paid) before the 31st of August 1781, to his Excellency M. de Hielmstierne, Knight of the Order of Danebrog, and President of the Society.

---

A paper has been lately communicated to the Royal Society by Richard Kirwan, Esq. F.R.S. describing the means of ascertaining the specific quantity of pure acid contained in any acid salt. In this paper the ingenious author has particularly determined the exact quantity of fixed air contained in different substances. By combining algebraical calculations with chemistry (a method that seems to be equally new and ingenious) he finds himself able to ascertain the 2500th part of a grain. The same philosopher is at present prosecuting experiments with a view to determine the specific proportion of phlogiston contained in the different metals.

M. Achard has lately communicated to the Royal Academy of Sciences at Berlin, a new method of exciting a degree of heat much superior to any hitherto produced, and this by means of a ſmall quantity of charcoal, or any other combuſtible ſubſtance. At the ſame time he read the deſcription of a machine for dephlogiſticating the air of apartments, and of courſe rendering them fitter for reſpiration.

---

At the anniverſary meeting of the Medical Society of London, on the 18th of January, the following gentlemen were elected officers of the Society for the year enſuing; Dr. Simmons, F. R. S. Preſident; Dr. Lettſom, F. R. S. Treaſurer; Dr. Hulme, Librarian; Dr. Kooſtray, Dr. Wood, and Mr. French, Secretaries. On this occaſion an oration in Latin, on the beſt means of proſecuting medical enquiries, was delivered by Dr. Simmons.

